# Dynamic associations between the respiratory tract and gut antibiotic resistome of patients with COVID-19 and its prediction power for disease severity

**DOI:** 10.1080/19490976.2023.2223340

**Published:** 2023-06-12

**Authors:** Yifei Shen, Wenxin Qu, Fei Yu, Dan Zhang, Qianda Zou, Dongsheng Han, Mengxiao Xie, Xiao Chen, Lingjun Yuan, Bin Lou, Guoliang Xie, Ruonan Wang, Xianzhi Yang, Weizhen Chen, Qi Wang, Yun Teng, Yuejiao Dong, Li Huang, Jiaqi Bao, Chang Liu, Wei Wu, Enhui Shen, Longjiang Fan, Michael P. Timko, Shufa Zheng, Yu Chen

**Affiliations:** a Department of Laboratory Medicine, The First Affiliated Hospital, Zhejiang University School of Medicine, Hangzhou, China; b Key Laboratory of Clinical In Vitro Diagnostic Techniques of Zhejiang Province, Hangzhou, China; c Institute of Laboratory Medicine, Zhejiang University, Hangzhou, China; d State Key Laboratory for Diagnosis and Treatment of Infectious Diseases, National Clinical Research Center for Infectious Diseases, Collaborative Innovation Center for Diagnosis and Treatment of Infectious Diseases, First Affiliated Hospital, College of Medicine, Zhejiang University, Hangzhou, China; e Institute of Bioinformatics, Zhejiang University, Hangzhou, China; f Departments of Biology and Public Health Sciences, University of Virginia, Charlottesville, VA, USA

**Keywords:** Dynamic association, COVID-19, antibiotic resistome, respiratory tract, gut, disease severity, prediction

## Abstract

The antibiotic resistome is the collection of all antibiotic resistance genes (ARGs) present in an individual. Whether an individual’s susceptibility to infection and the eventual severity of coronavirus disease 2019 (COVID-19) is influenced by their respiratory tract antibiotic resistome is unknown. Additionally, whether a relationship exists between the respiratory tract and gut ARGs composition has not been fully explored. We recruited 66 patients with COVID-19 at three disease stages (admission, progression, and recovery) and conducted a metagenome sequencing analysis of 143 sputum and 97 fecal samples obtained from them. Respiratory tract, gut metagenomes, and peripheral blood mononuclear cell (PBMC) transcriptomes are analyzed to compare the gut and respiratory tract ARGs of intensive care unit (ICU) and non-ICU (nICU) patients and determine relationships between ARGs and immune response. Among the respiratory tract ARGs, we found that Aminoglycoside, Multidrug, and Vancomycin are increased in ICU patients compared with nICU patients. In the gut, we found that Multidrug, Vancomycin, and Fosmidomycin were increased in ICU patients. We discovered that the relative abundances of Multidrug were significantly correlated with clinical indices, and there was a significantly positive correlation between ARGs and microbiota in the respiratory tract and gut. We found that immune-related pathways in PBMC were enhanced, and they were correlated with Multidrug, Vancomycin, and Tetracycline ARGs. Based on the ARG types, we built a respiratory tract-gut ARG combined random-forest classifier to distinguish ICU COVID-19 patients from nICU patients with an AUC of 0.969. Cumulatively, our findings provide some of the first insights into the dynamic alterations of respiratory tract and gut antibiotic resistome in the progression of COVID-19 and disease severity. They also provide a better understanding of how this disease affects different cohorts of patients. As such, these findings should contribute to better diagnosis and treatment scenarios.

## Introduction

The global rise in antibiotic resistance poses a major health threat and is responsible for more than 700 000 deaths annually.^1^ The antibiotic resistance crisis has increased the challenges of antibiotic treatment for bacterial infections,^2^ largely due to the selection of antibiotic resistance genes (ARGs) collection, in the human gut and respiratory tract. The human gut and respiratory tract are considered important reservoir of ARGs.^3,4^ A few current studies have explored the function of antibiotic resistome in the development of infection, suggesting possible relationships between the gut ARGs and infection.^5–8^ The composition of gut ARGs showed differences between patients with COVID-19 and healthy cohorts.^9,10^ As a major portal of entry for SARS-CoV-2, the human upper respiratory tract is inhabited by an abundant and complex microbiome.^11^ However, the composition of respiratory tract ARGs and their variation in patients with COVID-19 is largely unknown. In addition, little is known about the association between the respiratory tract ARGs and the gut ARGs, which is one of the most crucial questions regarding ARGs’ contribution to the health of patients with COVID-19.

Given that prior evidence suggested a close link between human gut and respiratory tract microbiome and COVID-19 disease severity,^12^ in the current study, we investigated the dynamic alterations in the composition of respiratory tract ARGs of patients with COVID-19 and its correlation with ARG composition in the gut. To this end, we recruited 66 COVID-19 patients at three stages of the disease (i.e., admission, progression, and recovery) and conducted metagenome sequencing in sputum and fecal samples. A total of 143 respiratory tract metagenomes, 97 gut metagenomes, and 66 peripheral blood mononuclear cell (PBMC) transcriptomes were sequenced and used to systematically investigate the similarities and differences between the gut and respiratory tract antibiotic resistome in intensive care unit (ICU) and non-ICU (nICU) patients at admission, progression, and recovery stages. Our findings reveal the dynamic alterations in the respiratory tract and gut ARGs during COVID-19 infection and explore their relationship between host immune response and microbiota. The findings of this study provide insights into the role changes in the respiratory tract and gut antibiotic resistome and immune system play at COVID-19 different disease severity and progression stages.

## Results

### Composition of the respiratory tract and gut antibiotic resistome in patients with COVID-19

**Figure 1.**
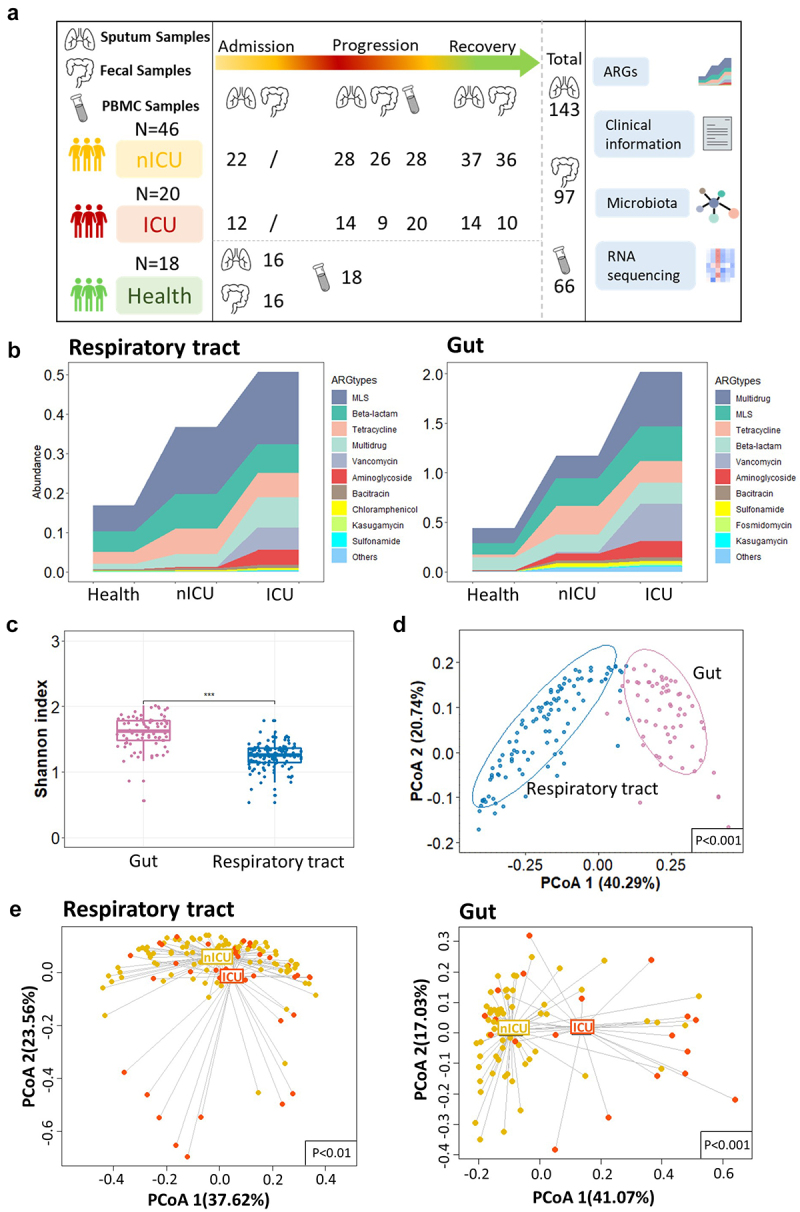
Composition of the respiratory tract and gut antibiotic resistome in patients with COVID-19.

To dissect the changes in the ARG composition of COVID-19 patients, we performed metagenome sequencing on 143 sputum and 97 fecal samples from 20 ICU and 46 nICU patients and 16 healthy people. The median ages of nICU patients, ICU patients, and healthy people were 51, 70, and 49 years, respectively; 39% and 100% of the patients in the nICU and ICU groups, respectively, had underlying comorbidities (Table 1). The sputum samples were collected from patients at three disease progression stages (admission, progression, and recovery), whereas the fecal samples were collected at two stages (progression and recovery) for the study (Figure 1a). We detected a total of 24 ARG types and 1244 ARG subtypes in the total sample population. After excluding unclassified ARG types or subtypes and 10% prevalence filtering, a total of 17 ARG types and 193 ARG subtypes were validated and subsequently analyzed in the current study. The top three most abundant ARG types identified in the respiratory tract are active against *Macrolide-lincosamide-streptogramin* (*MLS*), *Beta-lactam*, and *Tetracycline* and are present in both healthy individuals and patients with COVID-19 (Figure 1b). We found that among the top 10 most abundant ARG types in the respiratory tract, the relative abundances of *MLS*, *Vancomycin*, *Multidrug*, and *Aminoglycoside* increased significantly in patients with COVID-19 compared with healthy cohorts (*p*< .05) (Figure S1). ICU patients had higher relative abundances of *Multidrug*, *Vancomycin* and *Aminoglycoside* ARGs than nICU patients (Figure 1b). In the gut samples, the top three most abundant ARG types observed are *Multidrug*, *MLS*, and *Tetracycline*. We observed that among the top 10 most abundant ARG types, the relative abundances of *Multidrug*, *Tetracycline*, *Aminoglycoside*, *Fosmidomycin* and *Vancomycin* ARGs increased significantly in patients with COVID-19 compared with healthy cohorts (*p* < .05) (Figure S1). ICU patients had higher relative abundances of *Multidrug*, *Vancomycin* and *Fosmidomycin* ARGs than nICU patients (Figure 1b).

(a) Overview of the experimental design. (b) ARG types composition of ICU (*n* = 20) and nICU (*n* = 46) patients and a healthy cohort in respiratory tract and gut. (c) Comparison of alpha-diversity between respiratory tract and gut antibiotic resistome. * *p* < .05, ** *p* < .01, *** *p* < .001. (d) The first two axes of PCoA (Bray distance) for the beta-diversity of respiratory tract and gut antibiotic resistome. (e) The first two axes of PCoA (Bray distance) for the beta-diversity of ICU and nICU patient ARG types in the respiratory tract and gut. Group differences were tested using pairwise PERMANOVA. ICU: intensive care unit; nICU: non-ICU; PCoA: principal coordinate analysis; PERMANOVA: permutational multivariate analysis of variation.

**Table 1.** Basic demographics of the patients used in this study.

Characteristic	Non-ICU (*n* = 46)	ICU (*n* = 20)	Health (*n* = 18)
**Age, y**	51 (36.8–57.5)	70 (55.3–83)	49 (34–55.5)
**Male sex**	26 (56.5)	16 (80)	9 (50)
**Smoking history**	7 (15.2)	2 (10)	0 (0)
**Comorbidity**			NA
Diabetes	4 (8.7)	4 (20)	–
Cardiac disease	2 (4.3)	3 (15)	–
Liver disease	1 (2.2)	1 (5)	–
Lung disease	0 (0)	3 (15)	–
Any coexisting comorbidity	11 (23.9)	15 (75)	–
**Signs and symptoms**			NA
Fever	39 (84.8)	18 (90)	–
Cough	28 (60.9)	10 (50)	–
Sputum	12 (26.1)	7 (35)	–
**Laboratory results**			–
Leukocyte count, mm^3^	3.1 (1.9–5.9)	6.7 (3.6–12.6)	4 (3.5–5)
Lymphocyte count, mm^3^	1.0 (0.7–1.3)	0.5 (0.4–0.8)	1.8 (1.1–2.3)
Platelet count, mm^3^	181 (130–233)	182 (141.8–195.5)	243 (201–292)
Interleukin-6, pg/mL	17.3 (7–47.5)	46.6 (24–83.3)	2.1 (1.2–3.9)
Interleukin-10, pg/mL	3.7 (2.9–7.7)	6.9 (4.3–8.9)	0.6 (0.1–1.3)
**Treatments and outcomes**			NA
Antibiotic treatment	10 (21.7)	18 (90)	–
Quinolones (moxifloxacin)	6 (60)	6 (33.3)	–
Beta-lactam (piperacillin- tazobactam)	6 (60)	10 (55.6)	–
Beta-lactam (cefuroxime)	0 (0)	3 (16.7)	–
Glucocorticoids	33 (71.7)	20 (100)	–
Discharged	46 (100)	20 (100)	–

To investigate the diversity of respiratory tract and gut antibiotic resistome among patients with COVID-19, we calculated the Shannon index to measure the alpha diversity for each patient. We found that the Shannon index of the gut antibiotic resistome is much higher than that of the respiratory tract (*p* < .001) (Figure 1c). We also found that the beta-diversity was significantly higher in the gut antibiotic resistome than respiratory tract (*p* < .001) (Figure 1d). Furthermore, when we compared the beta-diversity of ICU and nICU patients, we found that there were significant differences between the ICU and the nICU patients in both the respiratory tract and gut antibiotic resistome (*p* < .01) (Figure 1e).

### Dynamic changes in respiratory tract antibiotic resistome and its association with disease severity

**Figure 2.**
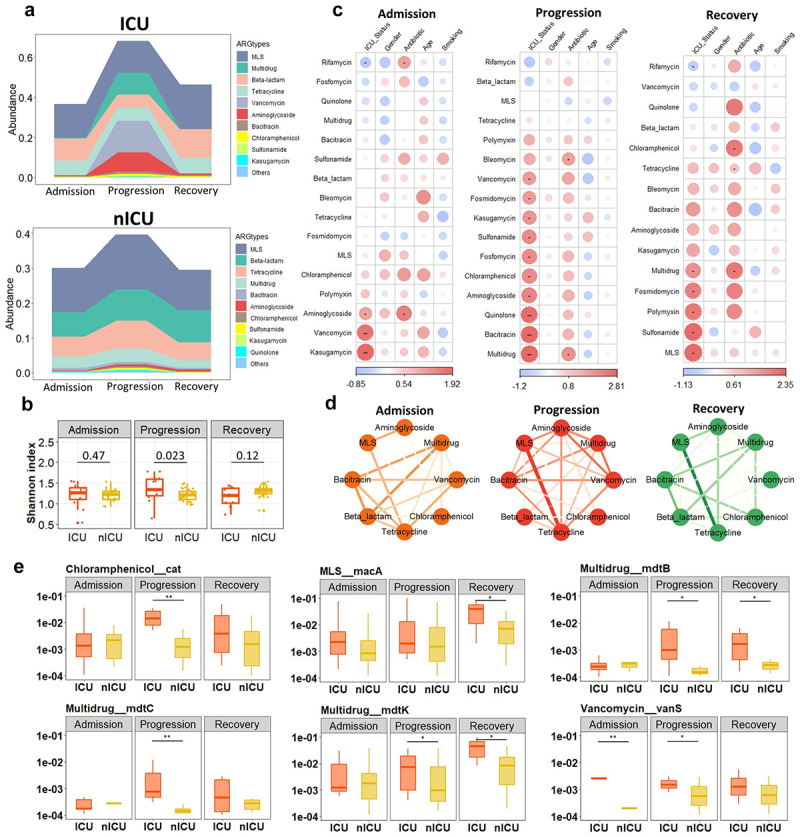
Dynamic alterations in respiratory tract antibiotic resistome and its association with disease severity.

We next examined the relative abundances of ARGs in sputum samples of patients at the admission, progression, and recovery stages to investigate the potential dynamic alterations in the respiratory tract antibiotic resistome over the time course of the disease. In the ICU patients, the total relative abundance of ARGs was significantly increased in the progression stage compared to the admission and recovery stages (*p* < .01) (Figure S2). Moreover, the relative abundances of *MLS*, *Multidrug*, *Vancomycin*, and *Aminoglycoside* ARGs were higher in the progression-stage samples compared to samples collected at the admission and recovery stages (Figure 2a), consistent with our previous findings (Figure 1b). In nICU patients, the total relative abundance of ARGs was significantly increased in the progression stage compared with the recovery stage (*p* < .05) (Figure S2). Further comparison of Shannon indices between ICU and nICU patients at different stages indicated that there was a significant difference in antibiotic resistome alpha diversity between ICU and nICU patients at the progression stage (*p* < .05) (Figure 2b).

(a) Respiratory tract ARG types composition of ICU (*n* = 20) and nICU (*n* = 46) patients at the admission, progression, and recovery stages. (b) Comparison of alpha-diversity between the respiratory tract ARG types of ICU and nICU patients at the admission, progression, and recovery stages. (c) Associations between respiratory tract ARG types and patient information at the admission, progression, and recovery stages. The color in the heatmap represents the regression coefficients estimated by multiple linear model regression analyses. * *p* < .05, ** *p* < .01, *** *p* < .001. (d) ARG types correlation networks constructed for the admission, progression, and recovery stages. Edge widths and colors are proportional to the strength of correlation. (e) Box plots show the relative abundances of ARG subtypes that showed significant differences between the ICU and nICU patients at the admission, progression, and recovery stages.

To further explore the differences in respiratory tract antibiotic resistome between ICU and nICU patients at different stages of disease progression, we performed multiple linear model regression analyses that included clinical information about patient ICU status, gender, antibiotic usage, age, and smoking history. At the admission stage, *Kasugamycin*, *Vancomycin*, and *Aminoglycoside* ARGs were significantly positively correlated with ICU status. In contrast, *Rifamycin* ARGs were negatively correlated with ICU status. Besides, at the admission stage, we found that *Rifamycin* and *Aminoglycoside* were positively correlated with antibiotic therapy (*p* < .05) (Figure 2c). At the progression stage, *Multidrug*, *Bacitracin*, *Quinolone*, *Aminoglycoside*, *Chloramphenicol*, *Fosfomycin*, *Sulfonamide*, *Kasugamycin*, *Fosmidomycin*, and *Vancomycin* ARGs were significantly positively correlated with ICU status (*p* < .05) (Figure 2c), and we observed that *Multidrug* and *Bleomycin* ARGs were positively associated with antibiotic therapy (*p* < .05) (Figure 2c). At the recovery stage, *Sulfonamide*, *Polymyxin*, *Fosmidomycin*, and *Multidrug* ARGs were significantly positively correlated with ICU status. In addition, we found that at the recovery stage, the relative abundances of *Multidrug* and *Chloramphenicol* ARGs were positively correlated with antibiotic therapy (*p* < .05) (Figure 2c). Based on these results, we found that *Kasugamycin, Vancomycin, and Aminoglycoside* ARGs were significantly positively correlated with ICU status in the admission and progression stages. Besides, the relative abundances of *Sulfonamide*, *Fosmidomycin*, and *Multidrug* ARGs were significantly positively correlated with ICU status in the progression and recovery stages. In addition, the results showed that patients’ gender, age, and smoking history did not have a significant influence on the abundance of respiratory tract antibiotic resistome (Figure 2c).

Next, to investigate the relationships between the ARG types at each stage, we performed a relative abundance correlation analysis (Figure 2d). Our results showed that *MLS* and *Tetracycline*, *MLS* and *Aminoglycoside*, *Bacitracin*, and *Multidrug* ARGs were significantly correlated with each other at all stages (*p* < .05) (Figure S2). *Tetracycline* ARGs were significantly positively correlated with *Vancomycin* ARGs both at the admission and progression stages (*p* < .05) (Figure S2). To determine the ARG subtypes involved in the observed changes, we compared the relative abundances of subtypes between ICU and nICU patients at the admission, progression, and recovery stages (Figure 2e). Among the *Chloramphenicol* types, the relative abundance of *Chloramphenicol_cat* increased considerably in ICU patients at the progression stage (*p* < .01). In the *MLS* types, the relative abundance of *MLS_macA* increased significantly in ICU patients at the recovery stage (*p* < .05). In the *Multidrug* types, the relative abundances of *Multidrug_mdtB*, *Multidrug_mdtC*, and *Multidrug_mdtK* were significantly increased in ICU patients at the progression stage (*p* < .05). In the *Vancomycin* types, the relative abundance of *Vancomycin_vanS* was significantly increased in ICU patients at the admission and progression stages (*p* < .05). Thus, the increased relative abundance of certain ARG types, such as *Multidrug* ARGs was related to alterations in many subtypes, whereas in *Chloramphenicol*, *MLS*, and *Vancomycin* ARGs only one subtype was responsible for the increase in the relative abundance of the types.

### Dynamic changes in gut antibiotic resistome of patients with COVID-19 and its association with respiratory tract antibiotic resistome

**Figure 3.**
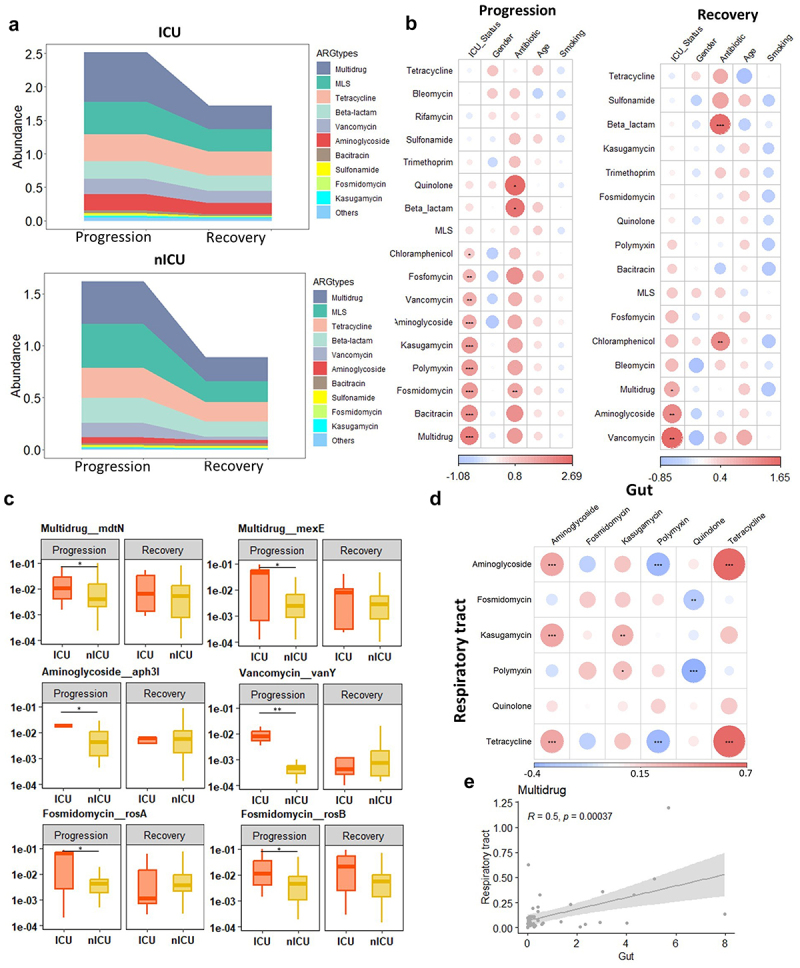
Dynamic alterations in gut antibiotic resistome of patients with COVID-19 and its association with respiratory tract antibiotic resistome.

To analyze the dynamic changes in the gut antibiotic resistome, we investigated the relative abundances of the ARG types in the gut during the progression and recovery stages. In ICU patients, the total relative abundance of ARGs was significantly increased in the progression stage compared to the recovery stage (*p* < .05) (Figure S3). Moreover, the relative abundances of *Multidrug* and *Fosmidomycin* ARGs were higher in the progression stage than in the recovery stage (Figure 3a). In nICU patients, no significant difference in the total relative abundance of ARGs was observed among samples collected at progression and recovery stages (Figure S3).

(a) Gut antibiotic resistome composition of ICU and nICU patients at the progression and recovery stages. (b) Associations between the gut antibiotic resistome and patient information at the progression and recovery stages. The color in the heatmap represents the regression coefficients estimated by multiple linear model regression analyses. * *p* < .05, ** *p* < .01, *** *p* < .001. (c) Box plots show the relative abundances of ARG subtypes that showed significant differences between the ICU and nICU patients at the progression and recovery stages. (d) Correlation between the gut and respiratory tract antibiotic resistome. (e) Correlation between the gut and respiratory tract antibiotic resistome of the *Multidrug*. The color in the heatmap represents the correlation coefficients estimated by Spearman correlation analysis. ICU: intensive care unit; nICU: non-ICU.

To further understand the differences in gut antibiotic resistome between the ICU and nICU patients at different disease stages, we also performed a multiple linear model regression analysis that included information about patient ICU status, gender, antibiotic usage, age, and smoking history. In the progression-stage samples, we found that *Multidrug*, *Bacitracin*, *Fosmidomycin*, *Polymyxin*, *Kasugamycin*, *Aminoglycoside*, *Vancomycin*, and *Fosfomycin* ARGs were significantly positively correlated with ICU status (*p* < .05). At the progression stage, we also observed that *Fosmidomycin*, *Beta-lactam*, and *Quinolone* ARGs were positively correlated with antibiotic therapy (*p* < .05) (Figure 3b). In the recovery stage, *Vancomycin*, *Aminoglycoside*, and *Multidrug* ARGs were found to be significantly positively correlated with ICU status (*p* < .05). In addition, at the recovery stage, we observed that *Beta-lactam* and *Chloramphenicol* ARGs were positively correlated with antibiotic therapy (*p* < .05) (Figure 3b). Based on these findings, we concluded that *Vancomycin*, *Multidrug*, and *Aminoglycoside* ARGs were positively correlated with ICU status in both the progression and recovery stages, and *Beta-lactam* ARGs were positively correlated with antibiotic therapy in both the progression and recovery stages.

Next, we compared the relative abundance of ARG subtypes between ICU and nICU patients at the progression and recovery stages to determine which ARG subtype contributed the most to the alterations in the gut antibiotic resistome (Figure 3c). In the *Multidrug* type ARGs, the relative abundance of *Multidrug_mdtN* and *Multidrug_mexE* subtypes were significantly increased in ICU patients at the progression stage (*p* < .05). In the *Aminoglycoside* type, the relative abundance of *Aminoglycoside_aph-3I* was significantly increased in ICU patients at the progression stage (*p* < .05). In the *Vancomycin* type, the relative abundance of *Vancomycin_vanY* was significantly increased in ICU patients at the progression stage (*p* < .01). In the *Fosmidomycin* type, the relative abundance of *Fosmidomycin_rosB* and *Fosmidomycin_rosA* subtypes were significantly increased in ICU patients at the progression stage (*p* < .05). Based on these results, we suggest that the increased relative abundance of the *Multidrug* and *Fosmidomycin* ARGs could be attributed to multiple subtypes, whereas in ARG types, such as *Vancomycin* and *Aminoglycoside*, only one subtype was responsible for the increased relative abundance.

To investigate the relationship between the antibiotic resistome of the respiratory tract and the gut, we conducted a correlation analysis based on the relative abundances of each ARG type. A significant positive correlation was revealed between the relative abundances of *Aminoglycoside*, *Kasugamycin* and *Tetracycline* ARGs in the respiratory tract and the gut (Figure 3d). In addition, we found that the *Multidrug* type was significantly increased in the ICU patients in both the gut and respiratory tract (Figure 6a). The relative abundance of *Multidrug* ARGs in the respiratory tract was significantly lower than that in the gut (Figure 1b). Based on the samples in which *Multidrug* ARGs were detected, a significant (*p* < .01) correlation was found between *Multidrug* ARGs in the respiratory tract and gut (Figure 3e).

### Relationship between respiratory tract, gut antibiotic resistome composition, and clinical indices

**Figure 4.**
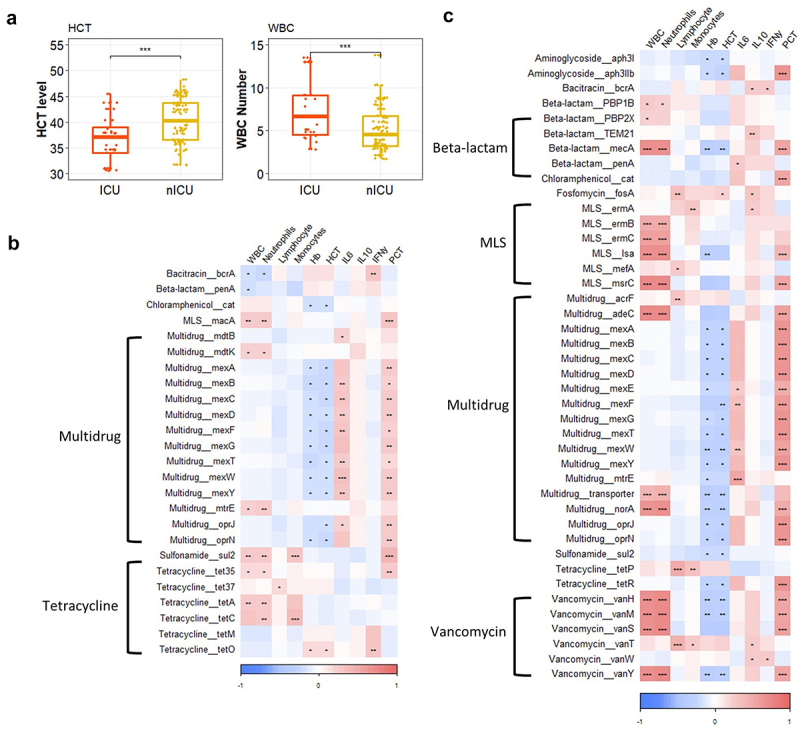
Relationship between respiratory tract, gut antibiotic resistome composition, and clinical indices.

We compared clinical indices in the ICU and nICU patients and found that the number of WBC was significantly higher in the ICU patients than the nICU patients (*p* < .05). In contrast, HCT levels were significantly higher in the nICU patients (*p* < .05) (Figure 4a). Both of these observations are consistent with results from previous studies.^13–15^ The levels of IL-6 and IL-10 were higher in the ICU patients compared with nICU patients and are both higher compared with healthy people. However, the number of lymphocytes was higher in healthy people compared with patients with COVID-19, and nICU patients have a higher lymphocyte count compared with ICU patients (Table 1). We further conducted a Spearman correlation analysis between clinical indices and the relative abundances of ARG subtypes, to explore the relationships between the clinical indices, and respiratory tract and gut antibiotic resistome of patients with COVID-19 (Figure 4b, c). In the respiratory tract, we found that IL-6 was positively correlated with the subtypes of *Multidrug* ARGs (*p* < .05), and PCT level, WBC and Neutrophil numbers were significantly positively correlated with the relative abundance of ARG subtype *MLS_macA* (*p* < .01), which was increased in the ICU patients at the recovery stage (Figure 2e). However, the Hb and HCT levels were significantly negatively correlated with the abundance of *Chloramphenicol_cat* and subtypes of *Multidrug* ARGs (*p* < .05), whereas the abundance of *Tetracycline_tetO* was positively correlated with the Hb and HCT levels (*p* < .05). The IFN-γlevel was positively correlated with the abundance of *Bacitracin_bcrA* and *Tetracycline_tetO* subtypes (*p* < .05) (Figure 4b). In the gut, PCT level was significantly positively correlated with the abundance of ARG subtypes *Multidrug_mexE* and *Vancomycin_vanY* (*p* < .001), both of which are higher in ICU patients at the progression stage (Figure 3c). IL-6 level was positively correlated with the abundance of many subtypes of *Multidrug* ARGs (*p* < .05). Moreover, WBC and Neutrophil numbers were positively correlated with the abundance of *Vancomycin_vanY* (*p* < .001), whereas the Hb and HCT levels were negatively correlated with the abundance of ARG subtypes *Aminoglycoside_aph3I* and *Vancomycin_vanY* (*p* < .05).

(a) Clinical indices in the ICU and nICU patients. (b) Relationship between clinical indices and relative abundances of ARG subtypes in the respiratory tract. (c) Relationship between clinical indices and relative abundances of ARG subtypes in the gut. The color in the heatmap represents the correlation coefficients estimated by Spearman correlation analysis. * *p* < .05, ** *p* < .01, *** *p* < .001. IL6: interleukin-6, IL10: interleukin-10, IFN-γ: interferon γ; WBC: white blood cell; PCT: procalcitonin.

### Co-occurrence patterns between disease-severity-related ARGs and microbial species in respiratory tract and gut

**Figure 5.**
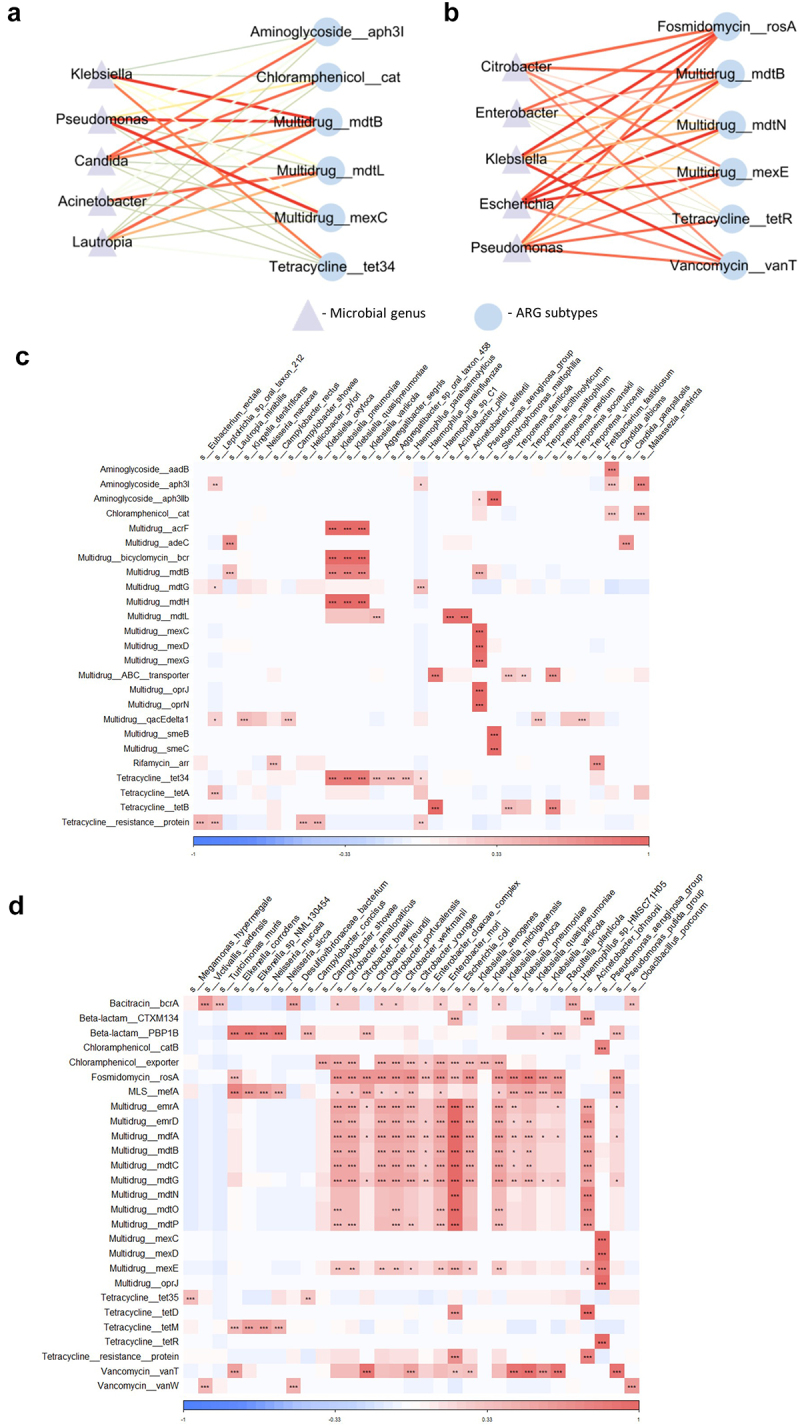
Co-occurrence patterns between disease-severity-related ARGs and microbial species in respiratory tract and gut.

To investigate whether there was a co-occurrence pattern between the disease severity-related ARGs and microbiota in the respiratory tract and gut, we conducted a correlation analysis based on the relative abundance of ARG subtypes and microbiota composition at the genus and species level. In the respiratory tract, at genus level, we found that the relative abundance of *Multidrug_mdtB* ARGs was positively associated with *Lautropia* (Figure 5a). Genera associated with pathogenicity, like *Lautropia*, in conditions of immune exhaustion were predominant in patients with severe infection.^16^ At the species level, we found that *Multidrug_mdtB* ARGs were positively associated with *Klebsiella pneumoniae*, which accounts for a higher proportion of hospital-acquired pneumonia (Figure 5c). Moreover, we found that subtypes of *Multidrug* ARGs were positively associated with *Pseudomonas aeruginosa*, which can cause infections in the blood or other parts of the body after surgery (Figure 5c). A prior study showed that patients with severe COVID-19 requiring ECMO had a very high rate of late-onset ventilator-associated pneumonia frequently caused by *P. aeruginosa*.^17^ This observation is consistent with our findings that the relative abundance of *Multidrug* ARGs is associated with disease severity (Figure 2a). We also observed that subtypes of *Aminoglycoside* and *Chloramphenicol_cat* ARGs were positively correlated with *Candida albicans*, a main cause of invasive fungal infections in critical care settings (Figure 5c). A prior study suggested a high prevalence of systemic candidiasis in severe COVID-19-associated pneumonia patients,^18^ which is consistent with our findings that the relative abundance of *Aminoglycoside* and *Chloramphenicol_cat* ARGs was higher in ICU patients and in the samples collected at the progression stage (Figure 2a-e).

ARG subtype correlation networks showing the Spearman correlation coefficients between disease-severity-related (a) respiratory tract and (b) gut antibiotic resistance genes (ARGs) and microbial genus. Edge widths and colors are proportional to the strength of correlation. The heatmap shows the Spearman correlation coefficients between disease-severity-related (c) respiratory tract and (d) gut ARGs and microbial species. The color in the heatmap represents the correlation coefficients estimated by Spearman correlation analysis. * *p* < .05, ** *p* < .01, *** *p* < .001.

In the gut, at the genus level, we found that the relative abundance of *Fosmidomycin_rosA, Vancomycin_vanT, and Multidrug_mexE* ARGs were positively correlated with *Escherichia*, *Citrobacter, and Pseudomonas* (Figure 5b). At the species level, we note that the relative abundance of *Fosmidomycin*_*rosA* and *Vancomycin_vanT* ARGs was positively correlated with *Pseudomonas putida*, whereas the relative abundance of *Multidrug_mexE* ARGs was positively correlated with *Pseudomonas aeruginosa*. We also observed that the relative abundance of *Fosmidomycin_rosA*, *Multidrug_mdtN*, and *Multidrug_mexE* ARGs were positively associated with *Escherichia coli* (Figure 5d). Prior studies showed that *E. coli* was positively correlated with COVID-19 disease severity.^19^ This observation is consistent with our finding that the relative abundances of *Fosmidomycin_rosA*, *Multidrug_mdtN*, and *Multidrug_mexE* ARGs were significantly increased in ICU patients at the progression stage in the gut (Figure 3c). Moreover, we found that at the species level, the relative abundance of *Multidrug_mdtB* ARGs was positively correlated with *K. pneumoniae* in both the respiratory tract and gut (Figure 5c, d).

### Respiratory tract and gut antibiotic resistome features are associated with immune response in PBMC

RNA sequencing was performed on PBMC samples of blood from ICU and nICU patients with COVID-19 at the progression stage, and from 18 healthy individuals to explore the relationships between respiratory tract ARGs, gut ARGs, and immune response-related gene expression. To explore this relationship, we carried out a Spearman correlation analysis between the gene set enrichment analysis (GSEA) score of each pathway and relative abundance of ARGs. In the respiratory tract ARGs, we found that the relative abundances of *Vancomycin_vanS* and *Multidrug_transporter* types were positively correlated with “PTK6 promotes HIF1A stabilization” (Figure S4). Moreover, the defensin-related pathways, such as “Defensins,” “Beta defensins,” and “Antimicrobial peptides,” were found to be significantly correlated with the relative abundance of subtypes of *Multidrug* and *Tetracycline* ARGs. In the gut ARGs, the results showed that the relative abundance of *Fosmidomycin_rosA* subtype, which was found to be significantly increased in ICU patients (Figure 3c), was positively correlated with “Synthesis of (16–20)-hydroxyeicosatetraenoic acids (HETE)” (Figure S4). This is consistent with the observation that infection with SARS-CoV-2 causes the imbalance of metabolites of arachidonic acid (AA) such as 20-HETE.^20^

### Respiratory tract and gut antibiotic resistome could accurately classify the disease severity of patients with COVID-19

**Figure 6.**
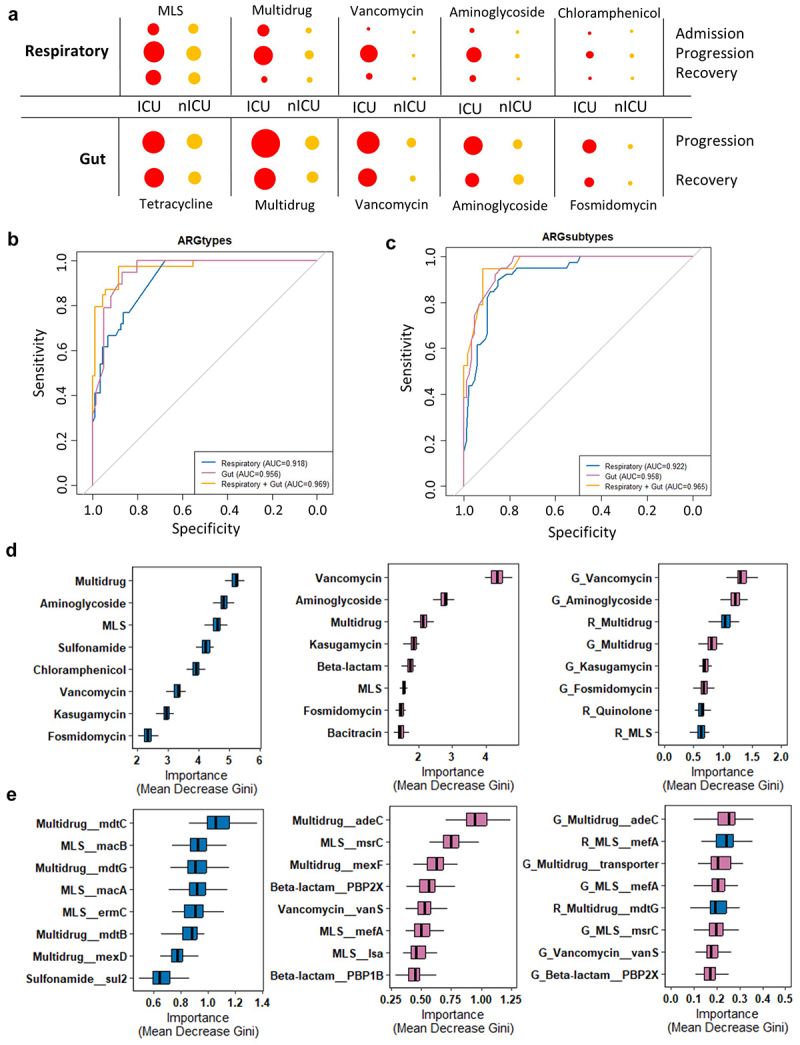
Respiratory tract and gut antibiotic resistance dynamics during COVID-19 progression and their diagnostic potential for disease severity.

To identify the antibiotic resistance characteristics associated with COVID-19 disease severity in the respiratory tract and gut, unsupervised random forest classification analysis using a leave-one-out cross-validation method was performed at the types and subtypes levels. We identified antibiotic resistance classifiers distinguishing ICU patients from nICU patients based on the respiratory tract ARGs, gut ARGs, and combined respiratory tract-gut ARGs in patients with COVID-19. At the ARG types level, the random forest classifiers based on the respiratory tract, gut, and combined respiratory tract–gut ARGs achieved area under the receiver operating characteristic curve (AUC) values of 0.918, 0.956, and 0.969, respectively (Figure 6b). The random forest classifier also identified the top ARG types characteristics of ICU and nICU patients. For the respiratory tract classifier, *Multidrug*, *Aminoglycoside* and *MLS* types were the top three characteristic ARGs, which was in line with our finding that relative abundances of *Multidrug*, *Aminoglycoside* ARGs in the respiratory tract were higher in the ICU patients (Figure 1b). For the gut classifier, *Vancomycin*, *Aminoglycoside*, and *Multidrug* types were the top three characteristic ARGs, which was in line with our finding that relative abundances of *Multidrug* and *Vancomycin* ARGs in the gut were higher in the ICU patients (Figure 1b). For the combined respiratory tract-gut classifier, *Aminoglycoside* and *Vancomycin* ARGs in the gut and *Multidrug* ARGs in the respiratory tract were the top three characteristic ARGs between ICU and nICU patients (Figure 6d).

(a) Graphical representation of major antibiotic resistance alterations during disease progression in the respiratory tract and gut. The size of the circle is broadly representative of the relative abundance of ARGs. ROC curves show the discriminative ability between ICU (*n* = 20) and nICU (*n* = 46) patients using the relative abundance of the respiratory tract, gut, and combined respiratory tract–gut antibiotic resistome at the (b) types and (c) subtypes level. The top eight important (d) types and (e) subtypes based on Gini importance according to random-forest classifiers based on the respiratory tract, gut, and combined respiratory tract–gut microbiomes. ICU: intensive care unit; nICU: non-ICU; ROC: receiver operating characteristic.

At the ARG subtypes level, the random forest classifiers based on the respiratory tract, gut, and combined respiratory tract–gut ARGs achieved area under the receiver operating characteristic curve (AUC) values of 0.922, 0.958, and 0.965, respectively (Figure 6c). The random forest classifier identified the top characteristic ARG subtypes between the ICU and nICU patients. In the respiratory tract, *Multidrug_mdtC*, *MLS_macB*, and *Multidrug_mdtG* were the top three characteristic ARG subtypes, which was consistent with the finding that the relative abundance of *Multidrug_mdtC* subtype in the respiratory tract was significantly higher in the ICU patients at the progression stage (Figure 2e). In the gut, *Multidrug_adeC*, *MLS_msrC*, and *Multidrug_mexF* were the top three characteristic subtypes. In the combined respiratory tract–gut classifier, *Multidrug_adeC*, and *Multidrug_transporter* in the gut and *MLS_mefA* in the respiratory tract were the top three characteristic subtypes between ICU and nICU patients (Figure 6e).

## Discussion

We have systematically examined the similarities and differences between respiratory tract and gut antibiotic resistome in patients with COVID-19 at different disease progression stages and with different disease severities in the ICU or not (nICU). Our data not only uncovered a shift of the respiratory tract and gut ARGs with the progression of COVID-19 but also demonstrated that relationships exist between ARG compositions and host immune response and microbiota in the respiratory tract and gut. The overuse and misuse of antibiotics has stimulated a more rapid emergence of ARGs in recent years.^21^ In our study, almost all of the ICU patients received empirical antibiotics, whereas there were 10 patients who received antibiotics in nICU group. We found that there was no significant difference in total relative abundance of ARG types in the respiratory tract and gut of patients who received antibiotics and nICU patients that never received antibiotics (Figure S5). Moreover, the relative abundance of ARGs in the respiratory tract and gut was higher in patients who never received antibiotics in the nICU group compared with healthy individuals (Figure S5).

Although it is difficult to determine whether the ARG features are causally correlated with the COVID-19 disease severity and progression, our data permit reasonable speculation on this matter. For example, we found that the relative abundances of *Vancomycin* and *Multidrug* ARGs in the respiratory tract and gut were significantly increased in ICU patients compared with nICU patients, and were higher in the progression stages in ICU patients. Moreover, we found that the relative abundance of *Vancomycin_vanS* subtype was significantly increased in both respiratory tract and gut in the COVID-19 patients, and was higher in the ICU patients (Figure S6), consistent with the results from a prior study.^9^ These results reveal a potential link between the relative abundance of *Vancomycin* and *Multidrug* ARGs, especially relative abundance of *Vancomycin_vanS* subtype with COVID-19 disease severity and progression. *Vancomycin_vanS* is a membrane-bound sensor histidine kinase that has been demonstrated to activate transcription of vancomycin-resistance genes.^22^

To explore the potential mechanism between ARGs and COVID-19 disease severity, we performed examination on several relationships. Bacterial co-infections are identified in patients with COVID-19 and are significant reasons for morbidity and mortality.^23^ Our study found that the relative abundance of *Multidrug_mdtB* subtype was positively correlated with *K. pneumoniae* in both the respiratory tract and gut. Moreover, we found that the relative abundance of *Multidrug_mexE* subtype was positively correlated with *P. aeruginosa*, which is characterized by intrinsic resistance to a variety of antimicrobial agents. This property results from the interplay between broadly specific drug efflux systems, including MexEF-OprN, and the low outer membrane permeability of *P. aeruginosa*.^24^ These results were in line with our finding that the relative abundance of *Multidrug_mexE* and *Multidrug_mdtB* subtypes was higher in ICU patients at the progression stage in the gut and respiratory tract, respectively (Figures 2e and 3c).

Previous evidence showed that IL6 and PCT levels had a highly significant association with COVID-19 disease severity.^25^ In our study, both were positively correlated with the relative abundance of *Multidrug* subtypes, which were found to be higher in the ICU patients both in the respiratory tract and gut. The increased immune response caused by viral infection may alter the respiratory tract microbiota, which can further regulate the local immune response.^26,27^ Taken together, these results provide a potential interpretation for the mechanism behind the ARGs and COVID-19 disease severity and progression. However, based on the current results, it is hard to unequivocally conclude whether higher ARGs, especially *Vancomycin_vanS* and *Multidrug_mdtB* abundances, caused changes in disease severity or whether the disease severity alterations caused higher ARGs abundances, especially *Vancomycin_vanS* and *Multidrug_mdtB*.

Our findings highlight that COVID-19 patients, especially those with severe disease, may be vulnerable to the accumulation of ARGs due to respiratory tract and gut dysbiosis,^12,28^ or an enhanced host immune response.^29^ Respiratory tract dysbiosis in deceased COVID-19 patients characterized by enrichment of conditional fungi such as *Candida* sp., which will impose considerable comorbidity and aggravate the disease.^30,31^ Gut dysbiosis in COVID-19 patients is characterized by enrichment of pathogenic bacteria like *Klebsiella* sp., the most common carriers of ARGs,^32^ which will result in bacterial coinfections. In addition, *K. pneumoniae* isolates obtained from COVID-19 patients have high antimicrobial resistance that accelerated the horizontal gene transfer via outer membrane vesicles, and increased the transfer of ARGs from pathogenic bacteria to commensal.^33,34^ The potential role of an immune balance is supported by the increased levels of inflammatory cytokines and their significant correlation with ARGs alteration. Our study found that *Vancomycin_vanS* in respiratory tract was positively correlated with “PTK6 promotes HIF1A stabilization” pathway, and HIF1A plays a key role in promoting SARS-CoV-2 infection and inducing pro-inflammatory responses to COVID-19.^35^ In conclusion, our study suggests that both the overgrowth of pathogenic bacteria and an increased immune response to SARS-CoV-2 infection might play a role in ARG accumulation.

The present study has several strengths. First, we were able to profile the respiratory tract and gut antibiotic resistome configuration of patients with COVID-19 and to the best of our knowledge this has not been previously investigated. Second, while a previous study mainly focused on the relationship between the antibiotic resistome and COVID-19 severity, we also examined the correlation between antibiotic resistome and COVID-19 disease progression. Finally, we compared, for the first time, the respiratory tract and gut antibiotic resistome as prognostic biomarkers for the COVID-19 severity. We also recognize that our study had several limitations. First, this was a single-center study, and, therefore, any generalization of our findings requires validation in other countries and/or ethnicities. Second, our current results did not allow us to derive a detailed mechanism or direct causal relationship between specific aspects of the antibiotic resistome and disease severity. Therefore, a more mechanistic investigation should be undertaken in the future. The current work provides a clear foundation for such exploration.

In summary, the present study shows that dynamic changes existed in the respiratory tract and gut antibiotic resistome of COVID-19 patients with different disease severity and progression, and there are significant changes in the composition of respiratory tract and gut ARGs between ICU and nICU patients. Our findings demonstrate that respiratory tract ARG composition, in particular, changes in the abundance of *Aminoglycoside*, *Vancomycin*, and *Multidrug* subtypes in COVID-19 patients, and gut ARG composition, in particular, changes in the abundance of *Multidrug*, *Vancomycin*, and *Fosmidomycin* subtypes, are correlated with disease severity. Our findings also suggest that respiratory tract and gut antibiotic resistome are closely connected to host immune response and respiratory tract and gut microbiota. Taken together, these novel ARG features provide valuable insight for the future development of protocols and tools for halting the continuing COVID-19 health crisis.

## Material and methods

### Study design and participants

A total of 66 patients with COVID-19 (46 nICU hospitalized patients and 20 ICU hospitalized patients) and 18 healthy individuals from the First Affiliated Hospital of Zhejiang University were enrolled in this study. Volunteers enrolled in this study varied in age between 13 and 96 years old. The ICU patients were included in the ICU group if they met one of the following conditions: 1) had respiratory failure and required mechanical ventilation, 2) entered into shock, and 3) had complicating nonfunction of other organs.^36^ Other hospitalized patients were included in the nICU group. We collected sputum and fecal samples from patients at three disease stages (admission, progression, recovery). The disease stages were defined according to a series of clinical symptoms, chest CT scans, laboratory indicators, and virological test results. Patients with the progression of pulmonary inflammatory lesions and higher levels of laboratory inflammation indicators were identified in the progression stage. Patients with improved clinical symptoms, respiratory function, absorption of pulmonary inflammation, and negative virological test results were identified in the recovery stage. In addition, PBMC samples were collected from 48 patients (28 nICU and 20 ICU) in progression stages. Healthy donors were all serologic negative for SARS-CoV-2, and without any comorbidities. All 18 healthy donors have RNA-seq data from PBMCs, and 16 of them have sputum and fecal ARG sequencing data. The study protocol was approved by the Ethics Committee of the First Affiliated Hospital, Zhejiang University School of Medicine, China (2021IIT A0239).

We obtained the patient epidemiological, clinical, and laboratory characteristics, as well as treatment and outcome data, from hospital electronic medical records. A trained team of doctors reviewed the data. Personal lifestyles, comorbidities, symptoms and signs, antibiotic treatment, laboratory indicators, and the progression and resolution of clinical illness were included as clinical data. Laboratory indicators included IL6, IL10, IFN-γ, TNF-α, and PCT levels and lymphocyte, neutrophil, and monocyte numbers. Patient’s smoking status was categorized as either current smoker or nonsmoker. Antibiotic treatment was categorized into used relevant antibiotics and non-used.

### Sample collection

Sputum from conscious patients and bronchial aspirates from unconscious patients were included in this study as the respiratory tract samples. Before peripheral blood mononuclear cells (PBMCs) extraction, blood samples were collected in specific collection tubes. PBMCs were then isolated and used for RNA-seq analysis. Fecal samples were collected in the hospital in a special sterile container and stored frozen on dry ice until used. Samples were then incubated in Dulbecco’s phosphate buffered saline, agitated for 15 min, and filtered through a 40-micron filter. During sampling, all medical personnel were equipped with personal protection equipment for biosafety level 3, including solid front-wrap around gowns, goggles, and N95 respirators.

### Library generation for PBMC transcriptome sequencing

Total RNAs were extracted from PBMC samples using a QIAamp RNA Blood Mini kit (Qiagen, Valencia, CA) according to the manufacturer’s instructions. Total RNA (1 μg) was used for sequencing library preparation, and the Illumina Total RNA-seq (H/M/R) Library Prep Kit was then used according to manufacturer’s instruction. We used an Agilent Bioanalyzer 2100 to examine library quality. The libraries were pooled together in equimolar amounts to a final concentration of 2 nM. The normalized libraries were denatured with 0.1 M NaOH (Sigma), and pooled denatured libraries were pair-end sequenced with a 150 bps read length on the Illumina NovaSeq 6000 platform.

### Library generation for metagenome sequencing

After the host cells were removed using a self-developed host removal kit, DNA was extracted from frozen sputum and fecal samples using a TIANamp Micro DNA kit (DP316, Tiangen Biotech) according to the manufacturer’s recommendations. Agilent 4200 TapeStation (Agilent Technologies) was used to assess DNA quality. DNA libraries were carried out by a standardized procedure for DNA fragmentation, end repair, adapter ligation, and PCR amplification. Agilent Bioanalyzer 2100 was used to assess library quality. Whole-genome shotgun sequencing of the sputum and fecal samples was performed on an Illumina HiSeq 2500 platform. All samples were paired-end sequenced with a 150-bp read length to a targeted data size of 5.0 Gb.

### RNA-seq data process

FASTQC^37^ was used to determine quality control (QC) metrics (base quality distribution) of the initial reads, and we then used NGS QC toolkits^38^ to trim low-quality reads. RNA-seq reads were mapped to the reference genome using Tophat.^39^ Following alignment, Cufflinks was used for transcript assembly^39^ and Cuffmerge^39^ was applied to merge transcripts generated from all sequencing samples. The output files were imported separately into Cuffdiff^39^ for further statistical analysis.

### Metagenome data process

Sequence reads were passed through the KneadData QC pipeline (http://huttenhower.sph.harvard.edu/kneaddata), which incorporates the Trimmomatic^40^ and BMTagger^41^ filtering and decontamination algorithms to remove low-quality read bases and reads of human origin, respectively: 1) trim non-human reads shorter than 50 nucleotides; 2) exclude samples with <500 000 microbial reads. Taxonomic profiling was performed using the MetaPhlAn2 classifier.^42^ The classifier relied on approximately 1 million clade-specific marker genes derived from >10 000 microbial genomes to unambiguously classify reads to taxonomies and yield the relative abundances of the taxa identified in the sample.

The ARG types and subtypes were annotated by ARGs-OAP v2.0 with default parameters.^43^ The abundance of ARG was calculated and normalized by the cell number in the research. We calculated and normalized ARG abundance using the following equation, expressed as ‘copies of ARG per prokaryote’s cell’,

(1) $$ARG\;abundance=\sum_{1}^{n}\frac{N_{ARG}\times L_{reads}/L_{ARG-ref}}{cell\;number}$$

where *n* is the number of ARG reference sequences belonging to an ARG type or subtype; *N*_*ARG*_ is the number of the ARG-like sequences aligned to one specific ARG reference sequence; *L*_*reads*_ is the sequencing length of metagenomic reads; *L*_*ARG-ref*_ is the length of the corresponding specific ARG reference sequences. The cell number was computed based on the following equation.^44^

(2) $$Cell\;\;number\;=\frac{\sum_{1}^{n}N_{16S\;\;Sequences}\times L_{reads}/L_{16S\;\;Sequences}}{\sum_{i=1}^{m}M_{i}\times a_{i}/A}$$

where *m* is the total taxa detected from the metagenomics dataset based on the extracted hypervariable region information; *M*_*i*_ represents the number of copies of taxon *i* from the CopyRighter database; *a*_*i*_ is the number of aligned hypervariable sequences of taxon *i* in the metagenomics dataset; *A* is the total number of aligned hypervariable sequences in all *m* taxa. ARG types or subtypes were excluded that were present in less than 10% of the samples; moreover, unclassified ARG types and subtypes were also excluded in the study.

### Statistical analysis

The alpha-diversity of the respiratory tract and gut antibiotic resistome was represented by the Shannon index in the study, which were calculated by the R software (ver.4.1.3) package “vegan.”^45^ The differences in alpha-diversity between groups were statistically measured using permutational multivariate ANOVA. Fisher exact tests and Mann–Whitney *U* tests were used to compare categorical variables and continuous variables, respectively. Beta diversity was used to evaluate differences in the antibiotic resistome among samples. Principal coordinates analysis (PCoA) on Bray–Curtis distance was performed to obtain visual representations. This analysis was implemented in the R “vegan” package^45^ and visualized using scatter plots. We then performed a multiple linear model regression analysis to investigate associations between ARG types and clinical information in the respiratory tract and the gut. Network analysis was used to explore the co-occurrence of ARGs in the COVID-19 patients based on Spearman correlation analysis. Networks were visualized and analyzed using Cytoscape.^46^ Spearman correlation analysis was also performed to investigate the relationship between clinical indices, immune-related pathways, and ARG subtypes, the association between gut and respiratory tract ARGs, and the relationship between ARGs and microbial genus and species. Correlation coefficients and significant P-values were computed using R software.

### Identification of ARG markers for predicting COVID-19 severity based on respiratory tract and gut metagenome data

To identify ARG markers that distinguish samples from ICU and nICU patients, classification models were performed based on ARG types and subtypes profiles using random-forest models. The models were validated by 10-fold stratified cross-validation testing. In each test, the accuracy of the model was examined by receiver operating characteristic. We conducted two steps in the random-forest model. In the first step, the model was constructed using each of the two antibiotic resistance profiles (i.e., respiratory tract and gut) independently. All the features were taken to conduct a random-forest function using the indicated parameters (500 trees, balanced class weight) and to compute the “feature importance” (Mean Decrease Gini). The optimal number of features was determined using the recursive feature elimination method.^47^ In the second step, the combination model was constructed by features selected from the separate models (respiratory tract and gut). The features were then selected by the recursive feature elimination method.^47^ We used the R software (ver4.1.3) package “randomForest” to perform all analyses.

## Supplementary Material

Supplemental Material

## Data sharing

The raw metagenomics sequencing dataset was deposited in the Sequence Read Archive (SRA) under BioProject accession PRJNA950577. Access will be granted after an appropriate request to corresponding author.
