# Supplementary figures and images for: Dynamic associations between the respiratory tract and gut antibiotic resistome of patients with COVID-19 and its prediction power for disease severity
Source: Gut Microbes. 2023 Jun 12;15(1):2223340. doi: 10.1080/19490976.2023.2223340 (PMC10262814; doi:10.1080/19490976.2023.2223340)

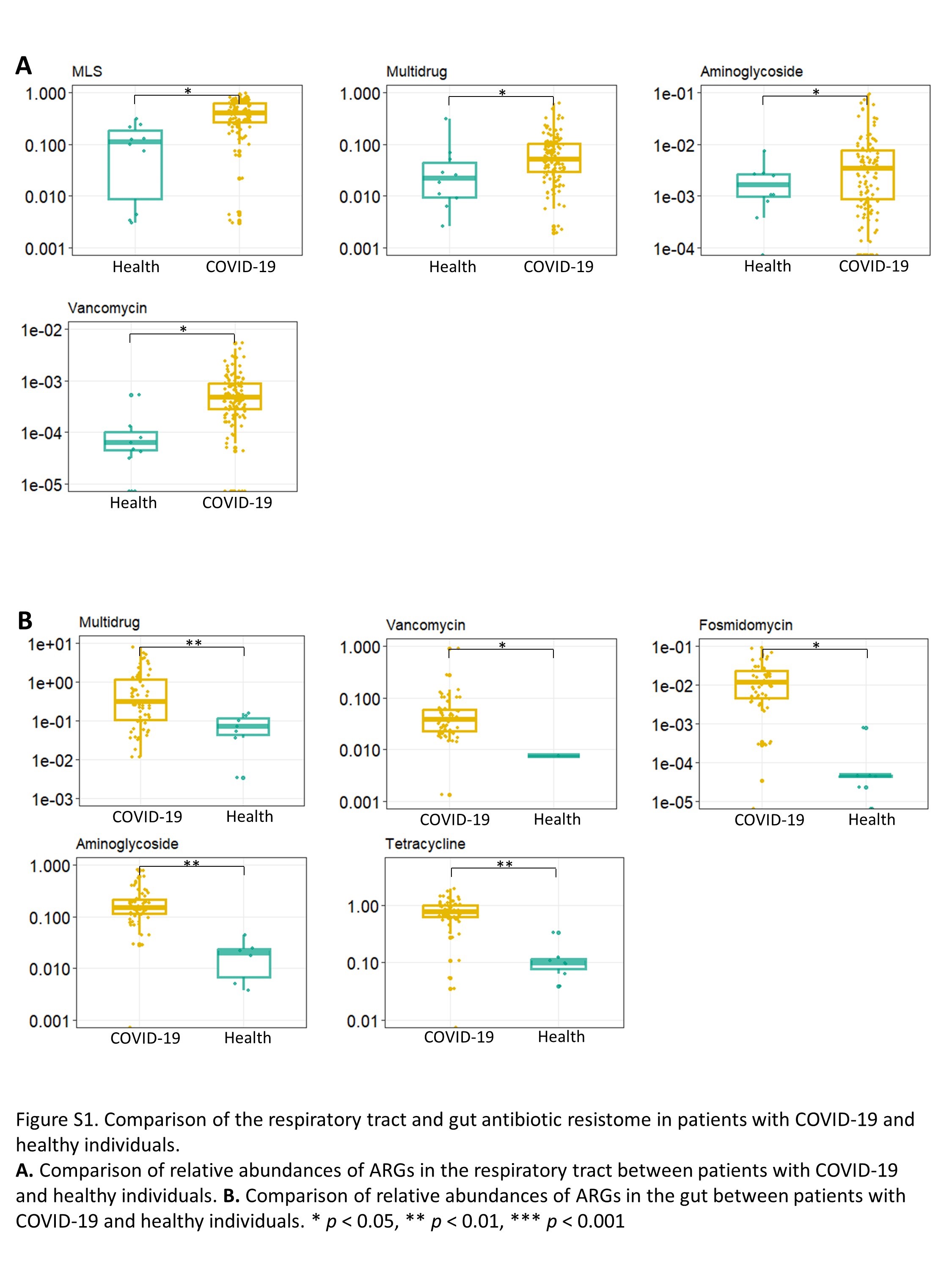

Supplement: Supplemental Material [file KGMI_A_2223340_SM4312.zip › Supplementary figures/Figure S1.JPG]

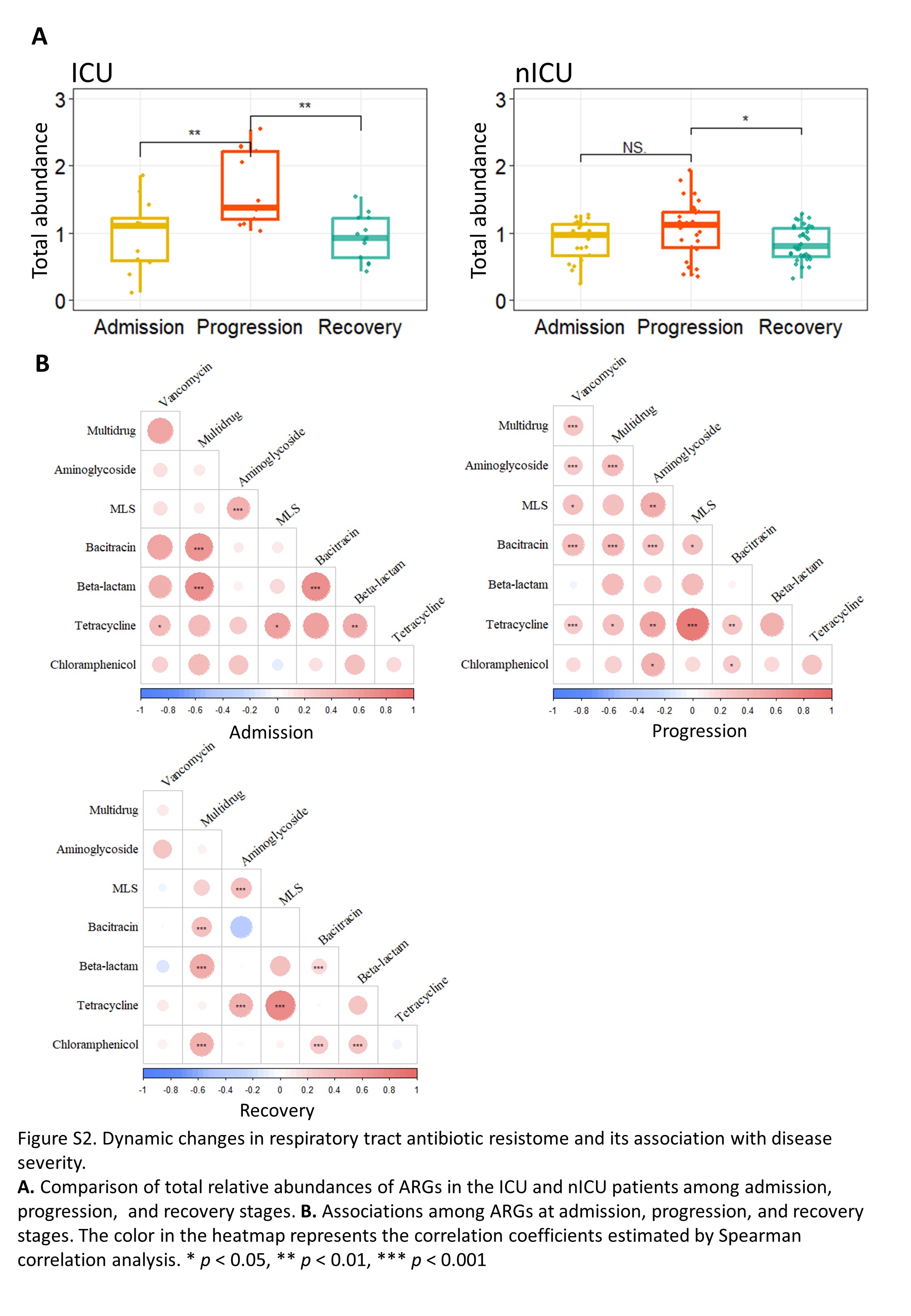

Supplement: Supplemental Material [file KGMI_A_2223340_SM4312.zip › Supplementary figures/Figure S2.JPG]

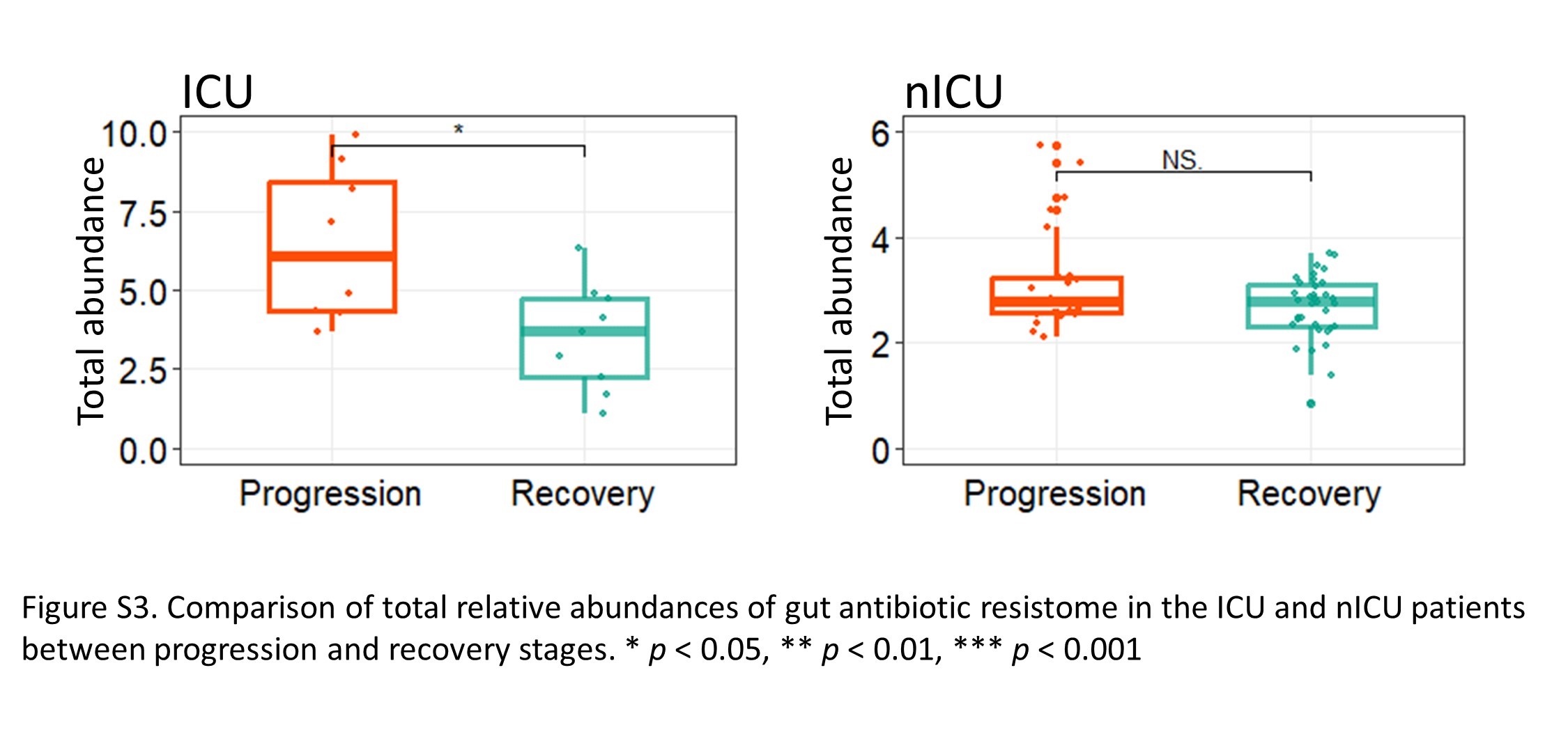

Supplement: Supplemental Material [file KGMI_A_2223340_SM4312.zip › Supplementary figures/Figure S3.JPG]

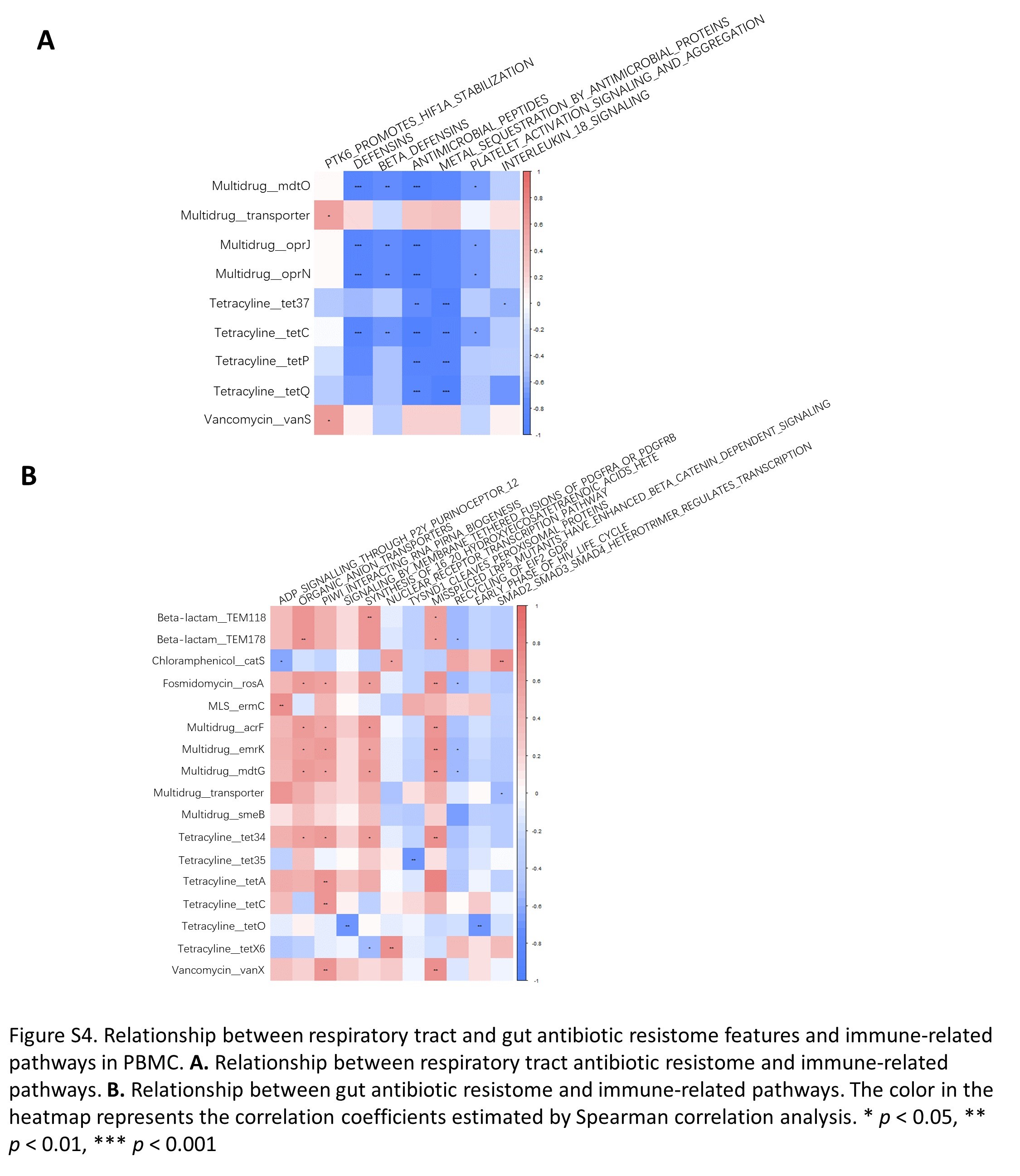

Supplement: Supplemental Material [file KGMI_A_2223340_SM4312.zip › Supplementary figures/Figure S4.JPG]

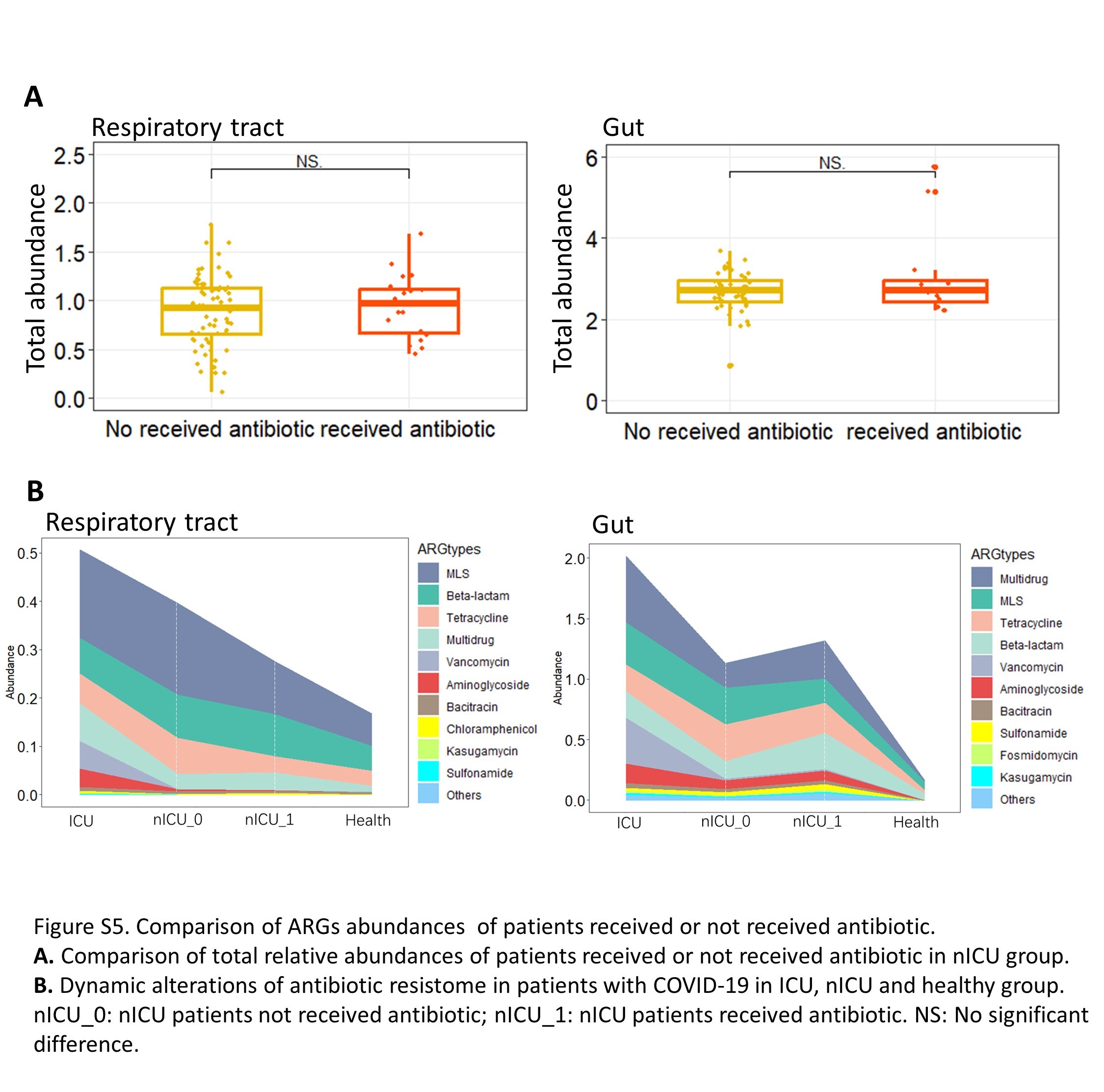

Supplement: Supplemental Material [file KGMI_A_2223340_SM4312.zip › Supplementary figures/Figure S5.JPG]

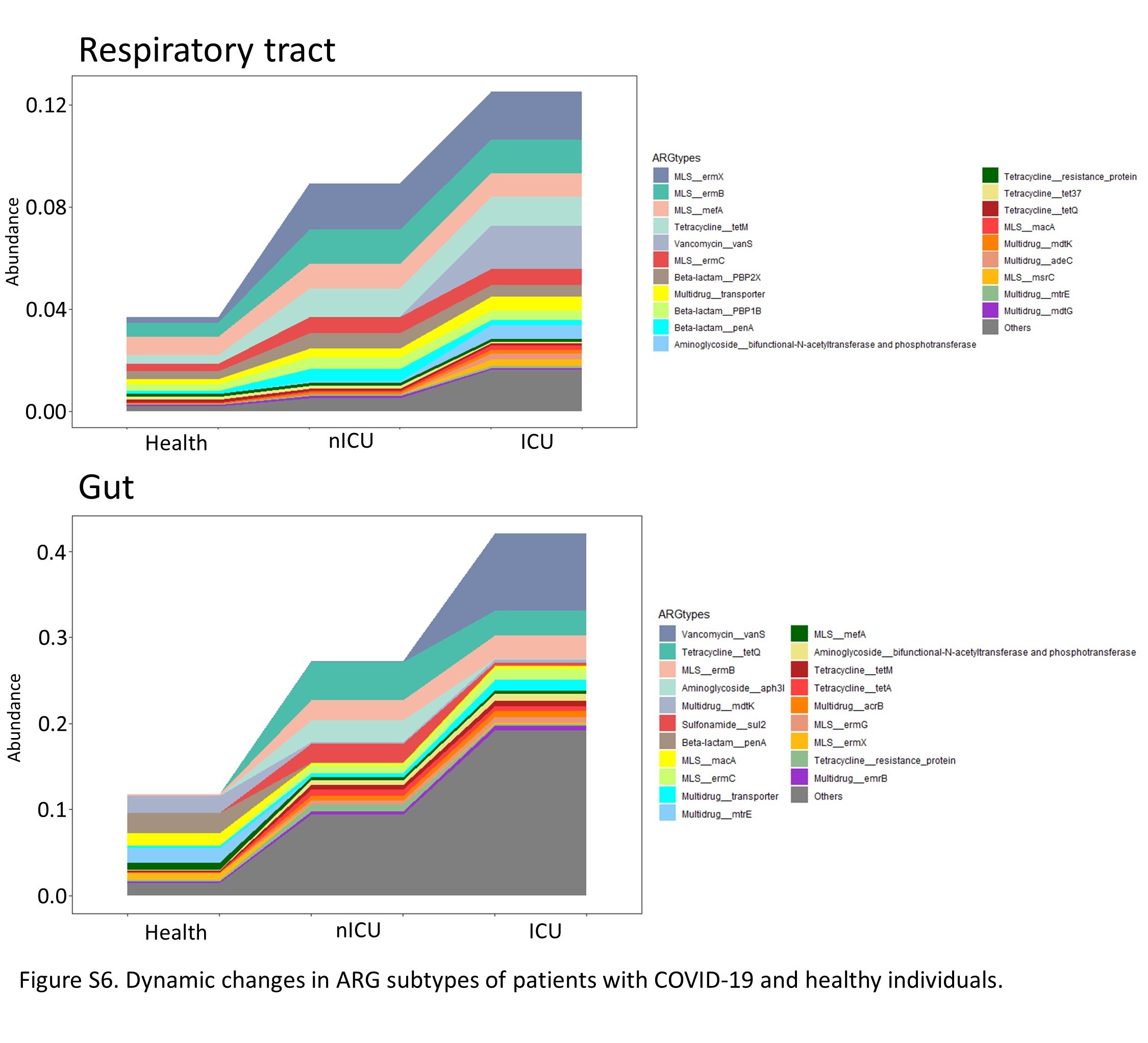

Supplement: Supplemental Material [file KGMI_A_2223340_SM4312.zip › Supplementary figures/Figure S6.JPG]
